# P3HT:PCBM blend films phase diagram on the base of variable-temperature spectroscopic ellipsometry

**DOI:** 10.3762/bjnano.9.102

**Published:** 2018-04-05

**Authors:** Barbara Hajduk, Henryk Bednarski, Bożena Jarząbek, Henryk Janeczek, Paweł Nitschke

**Affiliations:** 1 Centre of Polymer and Carbon Materials, Polish Academy of Sciences, 34 Marie Curie-Skłodowska str., 41-819 Zabrze, Poland

**Keywords:** non-linear optics, organic semiconductors, spectroscopic ellipsometry, theoretical modeling, thin films

## Abstract

In this work we present an in-depth study of the how the composition of poly(3-hexylthiophene) (P3HT):[6,6]-phenyl-C_61_-butyric acid methyl ester (PCBM) blend films influences their phase transitions using variable-temperature spectroscopic ellipsometry. We demonstrate that this non-destructive method is a very sensitive optical technique to investigate the phase transitions and to determine the glass transition temperatures and melting crystallization points of the P3HT:PCBM blend films. By analyzing the influence of the temperature *T* on the raw ellipsometric data, we have identified a high sensitivity of the ellipsometric angle Δ at a wavelength of 280 nm to temperature changes. Characteristic temperatures determined from the slope changes of the Δ(*T*) plot appeared to be very good guess values for the phase transition temperatures.

## Introduction

The organic semiconductor poly(3-hexylthiophene) (P3HT) and the fullerene derivative [6,6]-phenyl-C_61_*-*butyric acid methyl ester (PCBM) are extensively studied organic materials because of their important practical applications in organic electronics, especially in organic photovoltaic devices (OPV devices) [1–3]. Their properties are widely reported in literature [4–12]. Usually the OPV devices are constructed as sandwich structures with active layers located between cathode and anode. The most extensively studied and characterized OPV devices are bulk solar cells, with the donor P3HT and the acceptor PCBM. Such solar cells can be fabricated from mixed P3HT and PCBM solutions deposited on transparent electrodes by spin-coating [13–14]. Besides, bulk solar cells based on P3HT and PCBM blends became the benchmark for comparisons with maximum values of reported power conversion efficiency ranging from 5% to 8%. However, frequently reported mean values of OPV device efficiencies range from 3% to 5%. An important factor which influences OPV devices performance is the morphology of their active layers, e.g., the spatial order of the polymer backbone in the devices based on polymer–fullerene layers. For this reason, the morphology of the active layer in OPV devices has been intensively studied [15–16]. In general, it can be modified by the blend composition [17], the type of solvent used in processing [18], the use of chemical additives to the solution [19], and diverse post-deposition treatments, e.g., heat treatment [20–21]. One large branch of studies on OPV devices based on thin films of polymer:fullerene blend active layers deals with the optimization of their power conversion efficiency by applying thermal annealing [8]. Remarkably, it has been proven that the power conversion efficiency of OPV devices can be also optimized using thermal treatment related to the phase transition temperature. For instance, Pearson et al. [22] demonstrated that the most efficient devices were heated above the upper apparent glass transition temperature (*T*_g_) of P3HT:PCBM blends. They also related the value of the optimum annealing temperature for a given sample to their content of PCBM. What is important, these results were confirmed using two different experimental methods that allow for the determination of *T*_g_. Namely, dynamic mechanical thermal analysis (DMTA) and spectroscopic ellipsometry (SE). Moreover, with both techniques they detected two *T*_g_ transitions in thin films of P3HT:PCBM for a certain range of PCBM content. SE is a non-destructive and very sensitive technique for thin films investigations [23–25]. Generally, ellipsometry measures a change in the polarization of reflected light. The change of light polarization is described by ellipsometric angles Ψ and Δ. Typically, ellipsometric investigations allow one to determine optical parameters, i.e., the refractive index *n* and the extinction coefficient *k*, or equivalently the complex dielectric function ε ≡ (*n* + i*k*)^2^ ≡ ε_1_ + iε_2_, as well as the film thickness *d*. Variable-temperature spectroscopic ellipsometry additionally explores dependence of these quantities on the temperature *T*. This technique is sensitive to phase transitions because they are accompanied by a change in the volume expansion coefficient, which can be detected as change in the slope of *d*(*T*) plots [26]. Variable-temperature spectroscopic ellipsometry has been applied to study thin films of polymers such as polystyrene (PS) [27–31], poly(α-methylstyrene) [32], poly(methyl methacrylate) (PMMA) [33–34] and polyester [35], but also conjugated polymers such as polyfluorenes [36–38], quinoxaline and carbazole-based copolymers [39–40] and conjugated polymer:fullerene blends [23,41–42]. Frequently, the identification of the phase transition temperatures can be performed using the raw ellipsometric data [28,41,43]. However, almost always such an approach is verified for a particular material by an appropriate analysis of *d*(*T*) [22]. For example, Müller et al. [26] revealed the *T*_g_ profile of thin polymer films in depth and noticed that *T*_g_ can be detected directly using tanΨ. Also, Grohens et al. [44] compared the temperature dependence of the thickness and refractive index with cosΔ(*T*). In this work, we present an in-depth study of the influence of the composition of P3HT:PCBM blend films on their phase transitions using variable-temperature spectroscopic ellipsometry. We demonstrate that this non-destructive method is a very sensitive optical technique to investigate the phase transitions and to determine the glass temperatures and melting crystallization points for the P3HT:PCBM blend films. By analyzing the influence of the temperature on the raw ellipsometric data, we have identified the high sensitivity of the ellipsometric angle Δ at 280 nm to the temperature changes. By comparing the temperature dependence of the film thickness with the corresponding dependence of tanΨ (850 nm) and Δ (280 nm), we show that raw data of Δ at 280 nm are most convenient to determine the phase diagram of P3HT:PCBM blend films.

## Experimental

**Figure 1:**
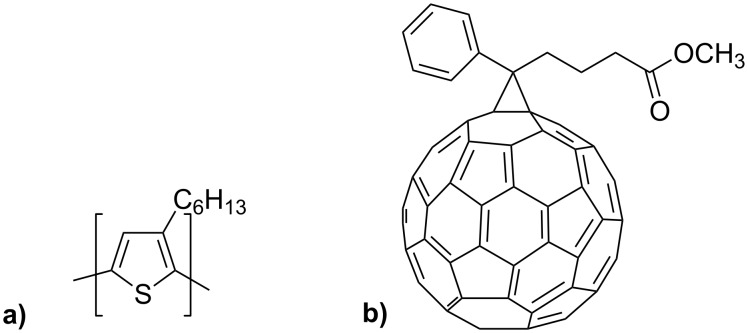
Structures of (a) P3HT and (b) PCBM.

Materials we have used are 95.7 wt % purity regioregular poly(3-hexylthiophene-2,5-diyl) M102-P3HT (Figure 1a) and above 99 wt % purity [6,6]-phenyl-C_61_-butyric acid methyl ester M111-PCBM (Figure 1b), supplied by Ossila.

Solutions of P3HT and PCBM in chlorobenzene were stirred for 24 h at a temperature of 60 °C. After that, the solutions were mixed together with different ratios of P3HT to PCBM from pure P3HT to pure PCBM, keeping a constant weight concentration of 20 mg/mL. The individual weight concentrations of ingredients were selected as indicated in Table 1. Thin films of P3HT, PCBM and P3HT:PCBM blend were deposited on opaque and transparent microscopic glasses through spin-coating at 2000 rpm. All samples were kept at 60 °C in the laboratory drier.

**Table 1:** Nominal composition of studied P3HT:PCBM blend films.

sample	1	2	3	4	5	6	7	8

P3HT [%]	100	85	70	60	50	35	20	0
PCBM [%]	0	15	30	40	50	65	80	100

Variable-temperature spectroscopic ellipsometry measurements have been performed using a SENTECH SE850E spectroscopic ellipsometer working in the spectral range of 240–2500 nm under the Spectra Ray 3 Software. The spectrometer is additionally equipped with a variable-temperature cell operating at low pressures and the temperature controller INSTEC mK1000. The cell construction allows for ellipsometric measurements at an incidence angle of 70° with a very precise temperature control through an electric heater and a liquid-nitrogen circuit.

We have applied the following measurement protocol for our variable-temperature spectroscopic ellipsometry investigations. Every sample was annealed at 250 °C for 2 min and quickly cooled down to −10 °C over a period of 3 min. It is important to note that thermal stability of P3HT and PCBM is high and their thermal degradation occurs only above 380 °C [45]. Then, ellipsometric measurements have been performed, during a heating cycle with a heating rate of 2 °C/min, in the UV–vis–NIR range and with 10 s intervals. All the measurements were performed under a pressure of 10^−1^ Torr.

The absorption measurements have been carried out at room temperature using a two-beam UV–vis–NIR spectrophotometer JASCO V-570.

Additionally, we have determined the glass transition temperatures using a DSC 2920 apparatus (TA Instruments, Newcastle, DE, USA), with aluminum sample pans. Thermal characteristics of the samples were obtained under nitrogen atmosphere (gas flow = 50 mL/min). The instrument was calibrated with high-purity indium and gallium standards. DSC measurements have been performed on powder materials (P3HT and PCBM) and on P3HT:PCBM (1:1) blend, which was scrapped off a glass substrate (thick layer, spin-coated with a spinning rate of 500 rpm).

## Results and Discussion

**Figure 2:**
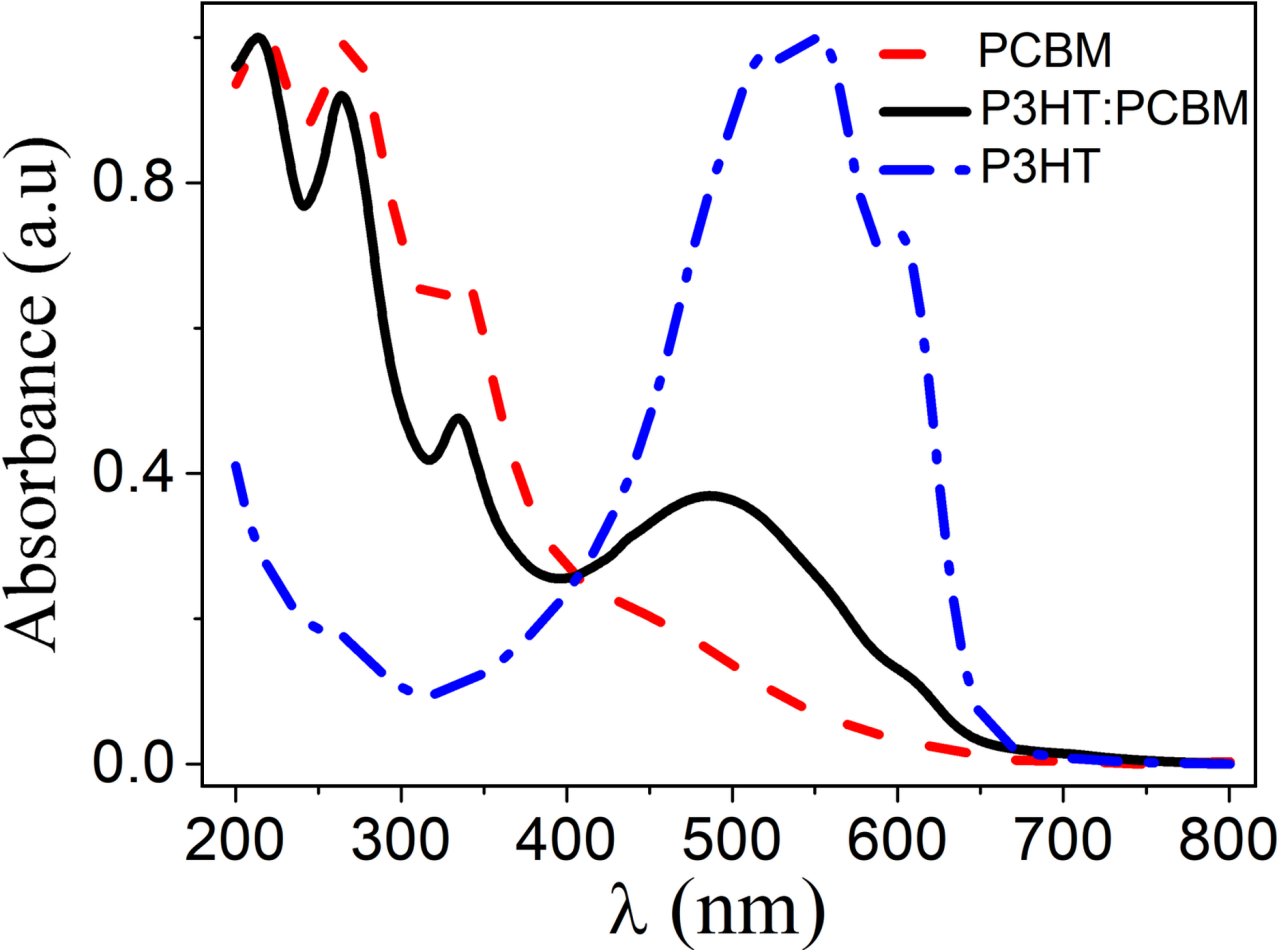
UV–vis absorbance spectra of P3HT, PCBM and P3HT:PCBM (1:1) thin films.

In Figure 2, the absorbance of thin films of P3HT, PCBM and P3HT:PCBM (1:1) is shown. It can be readily observed, that the absorbance in all these films disappears for wavelengths longer than λ = 750 nm. For this reason, in ellipsometric studies we can describe the studied films within the Cauchy optical model and determine their thickness as a function of the temperature at long wavelengths.

The Cauchy optical model parametrizes the spectral dependence of the refractive index *n* and the extinction coefficient *k* on the wavelength λ by the following relations:

[1]
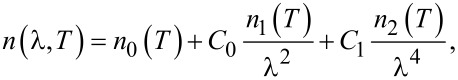


[2]
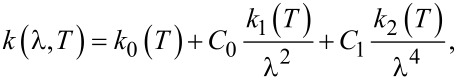


where the temperature-dependent parameters *n_i_* and *k_i_*, with *i* = 0, 1 and 2, are the model (fitting) parameters and the coefficients *C*_0_ and *C*_1_ are the numerical constants. It follows from the relations presented above, that at sufficiently long wavelengths both *n* and *k* become only temperature-dependent. Additionally, by taking into account the results presented in Figure 2, we can safely assume that *k*_0_(*T*) = 0 for λ > 750 nm.

**Figure 3:**
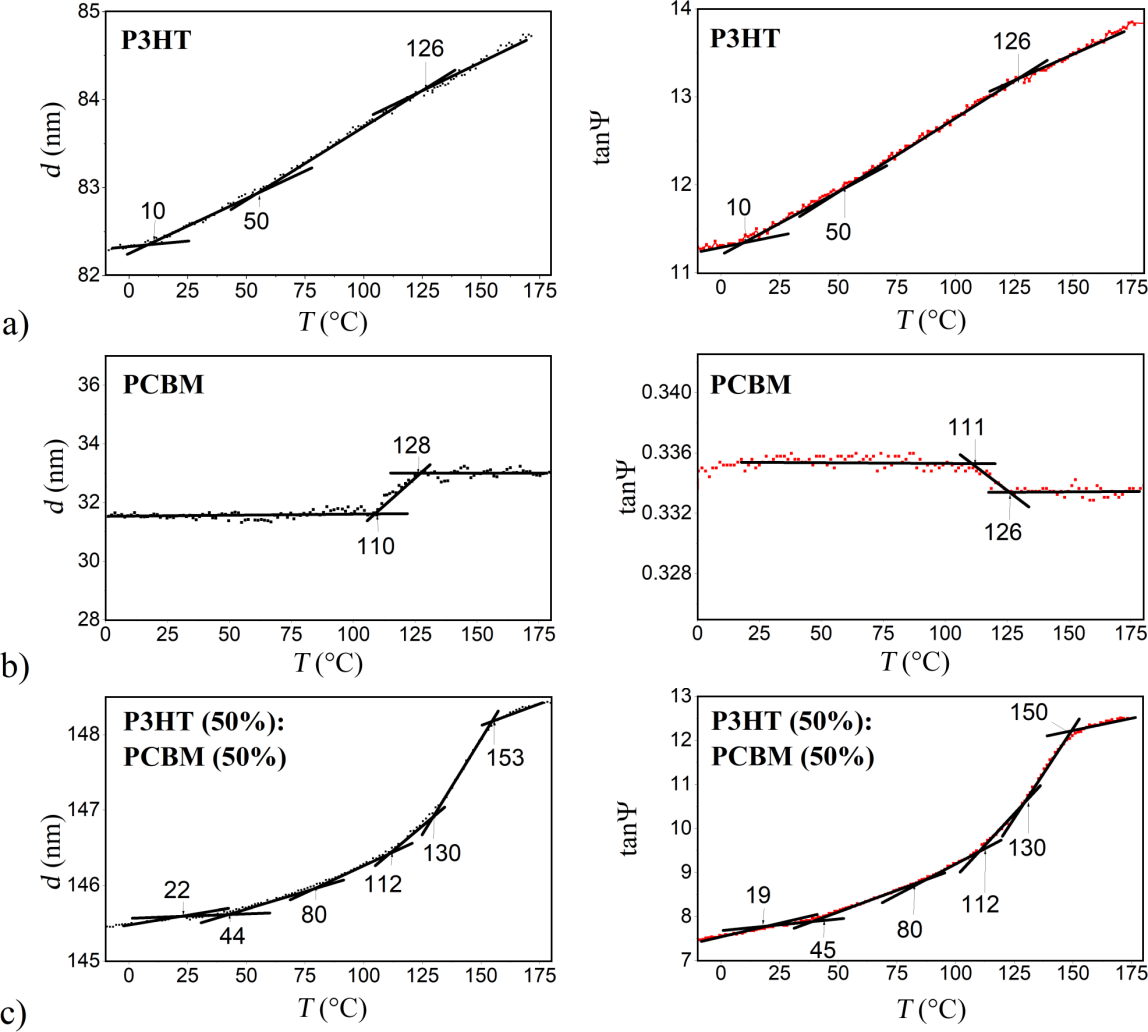
Thickness *d* and tanΨ as functions of the temperature for (a) P3HT, (b) PCBM and (c) P3HT:PCBM (1:1) thin films.

In Figure 3, thicknesses *d* as a function of the temperature for P3HT, PCBM and P3HT:PCBM (1:1) blend films are shown. At each measured temperature *T* the thickness was determined by fitting the Cauchy optical model to the experimental data using the SpectraRay 3 software from SENTECH Instruments.

For comparison, we have also presented in Figure 3 the corresponding temperature dependence of tanΨ taken at λ = 850 nm. Both plots, *d*(*T*) and tanΨ(*T*), are pairwise almost identical. This is because those materials are transparent at λ > 750 nm and their refractive indexes are weakly dependent on *T* in this spectral range. Regarding P3HT in Figure 3a, we can detect from a change in slope in the plots of *d*(*T*) and tanΨ(*T*) three characteristic temperatures at around 10, 50 and 126 °C. In Figure 3b, for PCBM, two characteristic temperatures are seen at 110 and 128 °C in the *d*(*T*) plot and at 111 and 126 °C in the tanΨ(*T*) plot. Significantly more characteristic temperatures can be detected in Figure 3c, for the P3HT:PCBM (1:1) film. The values are about 22, 44, 80, 112, 130 and 153 °C in the *d*(*T*) plot and about 19, 45, 80, 112, 130 and 150 °C in the tanΨ(*T*) plot. It is worthy of note, that the difference between corresponding characteristic temperatures detected in *d*(*T*) and tanΨ(*T*) plots do not excess 3 °C. Nevertheless, those results revealed also difficulties in a certain detection of the characteristic temperatures, at least in a few cases, e.g., those of 50 and 80 °C. For this reason, we have searched for more unambiguous indicators of thermal transitions. By comparing the temperature dependence of raw ellipsometric data of the P3HT:PCBM blend films with different PCBM content, we have found that Δ(*T*) taken at λ = 280 nm is the most convenient quantity for this purpose. Mainly because PCBM has a strong absorption band with the maximum located at around 260 nm.

**Figure 4:**
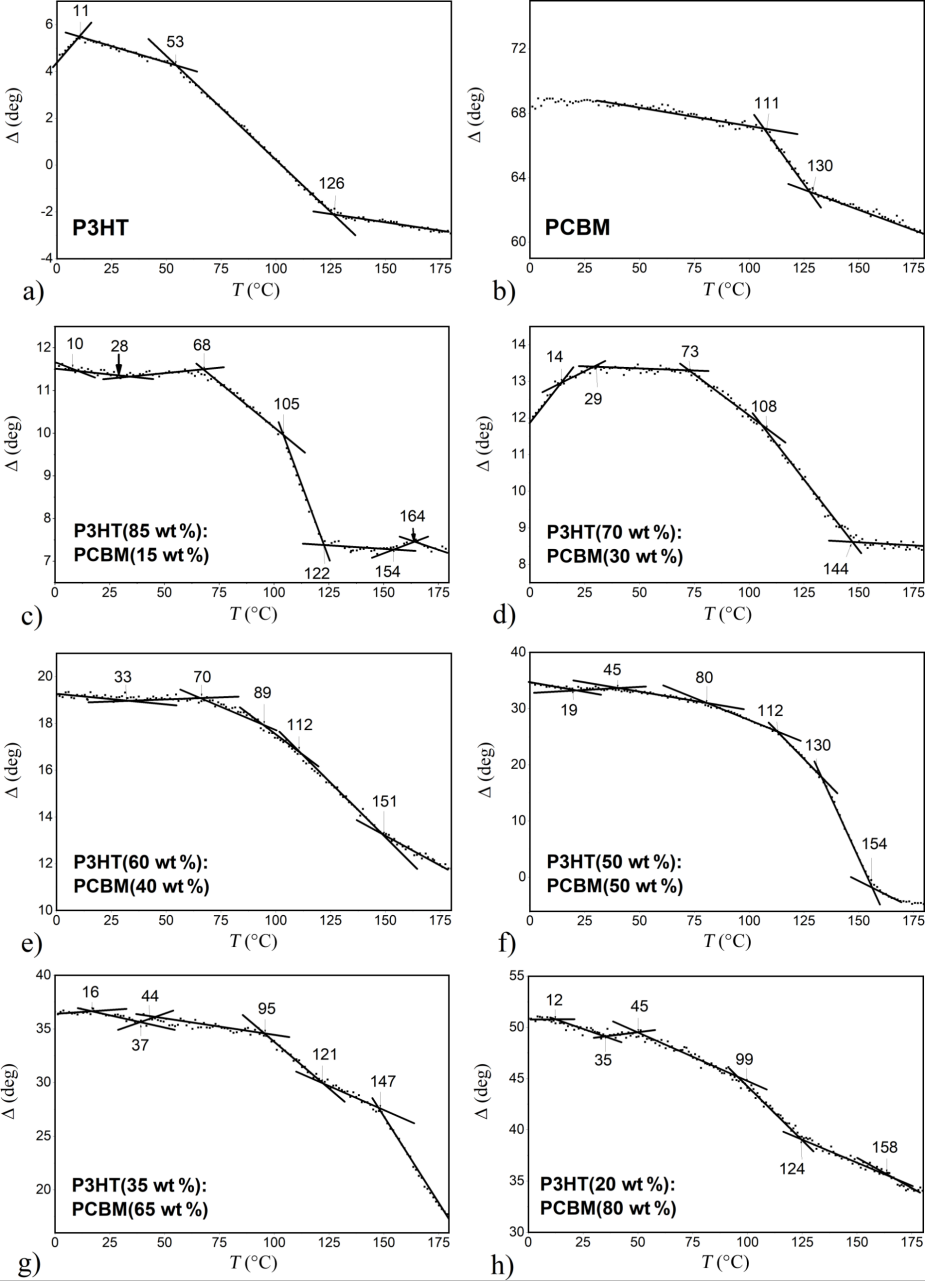
Δ at 280 nm as a function of temperature for P3HT:PCBM blend films with different polymer contents: a) P3HT, b) PCBM, c) 15, d) 30, e) 40, f) 50, g) 65 and h) 80 wt % PCBM.

**Figure 5:**
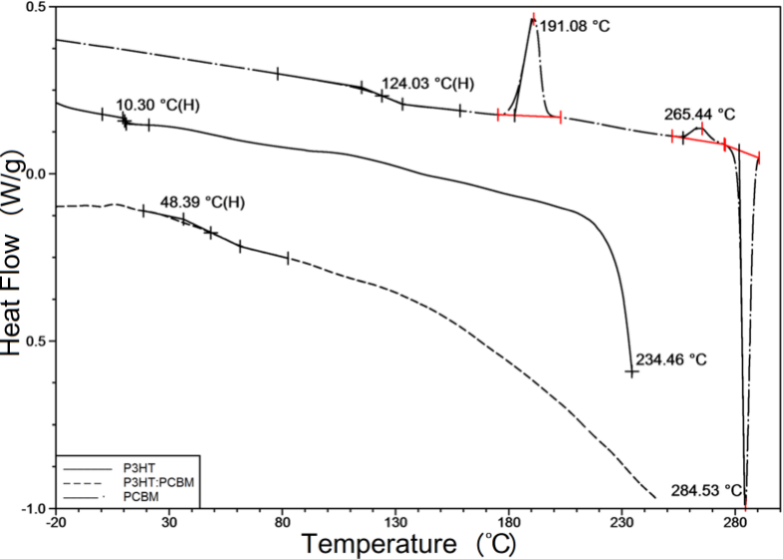
DSC plots with a heating rate of 20 °C/min for P3HT, PCBM and P3HT:PCBM (1:1), recorded with powder materials (P3HT and PCBM) and with the P3HT:PCBM (1:1) film scrapped off a glass substrate.

In Figure 4, Δ at λ = 280 nm as a function of the temperature is shown for the studied P3HT:PCBM blend films. The values of characteristic temperatures found in *d*(*T*) plots in Figure 3, fully correspond to the values detected in Figure 4, for P3HT, PCBM and P3HT:PCBM (1:1) films. We have verified that for all studied PCBM compositions the characteristic temperatures indicated in Figure 4, have been recovered on corresponding plots of *d*(*T*) and tanΨ(*T*), which are not shown here for clarity of presentation. In order to facilitate identification of the origin of all those thermal transitions we have performed DSC studies on powder materials (P3HT and PCBM) and on the P3HT:PCBM (1:1) film scrapped off a glass substrate. The results of the calorimetric measurements with a constant heating rate of 20 °C/min are shown in Figure 5.

**Figure 6:**
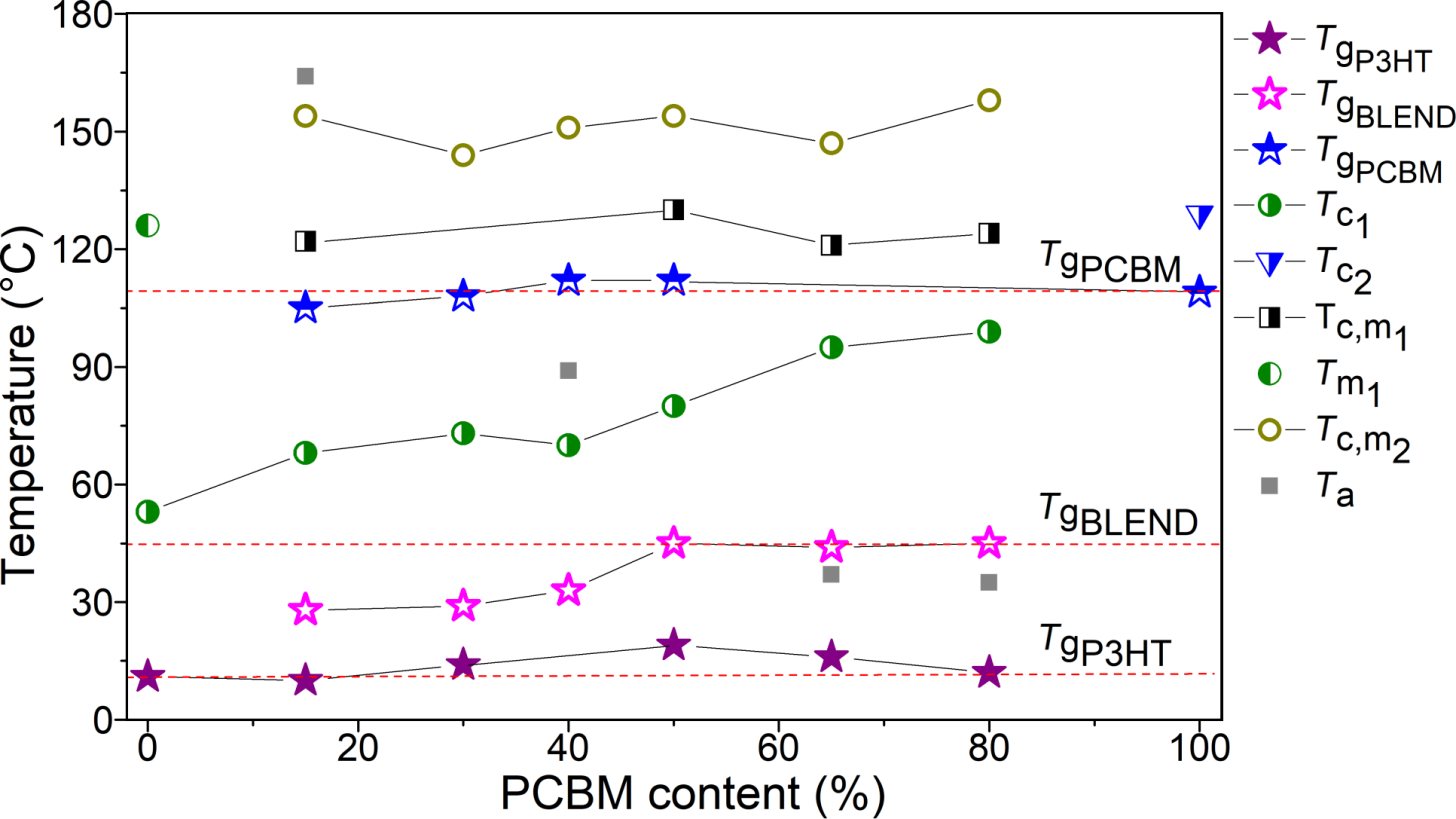
Phase diagram of P3HT:PCBM blends films. Symbols marked as *T*_gP3HT_, *T*_gBLEND_ and *T*_gPCBM_ denote the glass transitions of P3HT, P3HT:PCBM blend and PCBM phases. *T*_c1_, *T*_c2_, *T*_c,m1_, *T*_m1_ and *T*_c,m2_ are phase transitions related the P3HT crystallization, the PCBM cold crystallization, the melting of P3HT crystallization and/or crystallization of PCBM, melting of P3HT crystallization as well as the additional crystallization of PCBM, respectively. Symbols marked as *T*_a_ denote additional phase transitions.

A modest number of characteristic temperatures have been detected in Figure 5. Regarding P3HT, *T*_g_ is located at 10.30 °C and the beginning of the melting endotherm at around 215 °C is also visible. The temperature scan for the P3HT:PCBM sample, heated from −50 to 250 °C, reveals only the glass transition at 48.39 °C. The results for PCBM show *T*_g_ at 124.03 °C and two peaks of exothermic crystallization processes with maxima at 191.08 °C and at 265.44 °C. Also, the subsequent melting endotherm with a sharp minimum at 284.53 °C is visible. Having determined phase transitions from the DSC measurements, we return to our ellipsometric results. We can now identify in Figure 3 that glass transitions occurred in the P3HT, P3HT:PCBM (1:1) and PCBM films at temperatures of 10, 21 and 110 °C, respectively. The glass transition temperatures found by DSC were slightly higher than the corresponding temperatures determined with ellipsometry. This can be ascribed to different heating rates used in both experimental methods, and/or to the influence of films thickness on their phase behavior [22,46]. However, given that in our studies the thickness of the films varied over a wide range, between 30 nm (83 nm) and 150 nm, the latter cause seems to be more likely. In Figure 6, all characteristic temperatures for P3HT/PCBM blend films with different PCBM content, found in Figure 4 are shown.

This phase diagram can be rationalized as follows: We draw on it three horizontal straight lines passing through the glass transition temperatures of P3HT, P3HT:PCBM (1:1) and PCBM films. Now, it can be clearly seen that the glass transitions of pure materials are present even in our P3HT:PCBM blend films. This is consistent with the coexistence of the mixture as well as pure material phases in our samples. The glass transition temperatures, drawn with symbols marked as *T*_gBLEND_, increases with percentage PCBM content and can be ascribed to P3HT:PCBM mixtures. Their values are in good agreement with those reported in literature for P3HT:PCBM blend [45,47–48]. Transitions marked as *T*_c1_, lying below *T*_gPCBM_ should be ascribed to thermal transitions connected with the crystallization of P3HT, because this thermal transition is also present in P3HT. The identification of remaining transitions is ambiguous due to complex morphology of our P3HT:PCBM blend films. Nevertheless, it should be noted that the phase diagram determined by us is in a good agreement with that reported by Hopkinson et al. [47] obtained by dynamic mechanical thermal analysis.

## Conclusion

In this work we have presented an in-depth study of the influence of the composition of poly(3-hexylthiophene) (P3HT):[6,6]-phenyl-C_61_-butyric acid methyl ester (PCBM) blend films on their phase transitions using variable-temperature spectroscopic ellipsometry. We have investigated thin films of P3HT:PCBM blends deposited by spin-coating. Our studies included optical absorption spectroscopy, variable-temperature spectroscopic ellipsometry and differential scanning calorimetry. Based on the absorption spectra we determined the beginning of the spectral transparency region of our samples at λ = 750 nm. This allowed us to apply the Cauchy optical model to ellipsometric data analysis in the transparency region and determine the samples thickness *d* as a function of temperature *T*. The obtained *d*(*T*) plots were the base for the determination of phase diagrams. By analyzing the influence of temperature on the raw ellipsometric data, we have identified a high sensitivity of the ellipsometric angle Δ at a wavelength of 280 nm to temperature changes. Characteristic temperatures determined from the slope changes of the Δ(*T*) plot appeared to be very good guess values for the phase transition temperatures. We have identified characteristic temperatures originating from glass transitions and cold crystallization or melting of crystallization phases of P3HT, P3HT:PCBM mixture and PCBM. The clear advantages of this method are the possibility to directly monitor the phase behavior of P3HT:PCBM thin layers and to accurately estimate their characteristic temperatures. In addition, this method allows one to omit a time-consuming theoretical analysis of ellipsometric data and limits the measurements to a very narrow spectral range. This, in turn, complements the already known advantages of variable-temperature ellipsometry, such as less sensitivity to sample imperfections and very high sensitivity to sample thickness.
